# Effects of a lifestyle intervention during pregnancy to prevent excessive gestational weight gain in routine care – the cluster-randomised GeliS trial

**DOI:** 10.1186/s12916-018-1235-z

**Published:** 2019-01-14

**Authors:** Julia Kunath, Julia Günther, Kathrin Rauh, Julia Hoffmann, Lynne Stecher, Eva Rosenfeld, Luzia Kick, Kurt Ulm, Hans Hauner

**Affiliations:** 1 Else Kröner-Fresenius-Centre for Nutritional Medicine, Klinikum rechts der Isar, Technical University of Munich, Georg-Brauchle-Ring 62, 80992 Munich, Germany; 2 Competence Centre for Nutrition (KErn), Am Gereuth 4, 85354 Freising, Germany; 3 Institute of Medical Informatics, Statistics and Epidemiology, Klinikum rechts der Isar, Technical University of Munich, Ismaninger Straße 22, 81675 Munich, Germany

**Keywords:** Lifestyle intervention, gestational weight gain, diet, exercise, gestational diabetes, weight retention, childhood obesity, obesity prevention, pregnancy, obstetric outcomes

## Abstract

**Background:**

Excessive gestational weight gain (GWG) leads to obstetric complications, maternal postpartum weight retention and an increased risk of offspring obesity. The GeliS study examines the effect of a lifestyle intervention during pregnancy on the proportion of women with excessive GWG and pregnancy and obstetric complications, as well as the long-term risk of maternal and infant obesity.

**Methods:**

The GeliS study is a cluster-randomised multicentre controlled trial including 2286 women with a pre-pregnancy BMI between 18.5 and 40.0 kg/m^2^ recruited from gynaecological and midwifery practices prior to the end of the 12^th^ week of gestation in five Bavarian regions. In the intervention regions, four lifestyle counselling sessions covering a balanced healthy diet, regular physical activity and self-monitoring of weight gain were performed by trained healthcare providers alongside routine pre- and postnatal practice visits. In the control regions, leaflets with general recommendations for a healthy lifestyle during pregnancy were provided.

**Results:**

The intervention did not result in a significant reduction of women showing excessive GWG (adjusted OR 0.95, 95% CI 0.66–1.38, *p* = 0.789), with 45.1% and 45.7% of women in the intervention and control groups, respectively, gaining weight above the Institute of Medicine recommendations. Gestational diabetes mellitus was diagnosed in 10.8% and 11.1% of women in the intervention and control groups, respectively (*p* = 0.622). Mean birth weight and length were slightly lower in the intervention group (3313 ± 536 g vs. 3363 ± 498 g, *p* = 0.020; 51.1 ± 2.7 cm vs. 51.6 ± 2.5 cm, *p* = 0.001).

**Conclusion:**

In the setting of routine prenatal care, lifestyle advice given by trained healthcare providers was not successful in limiting GWG and pregnancy complications. Nevertheless, the potential long-term effects of the intervention remain to be assessed.

**Trial registration:**

NCT01958307, ClinicalTrials.gov, retrospectively registered October 9, 2013.

**Electronic supplementary material:**

The online version of this article (10.1186/s12916-018-1235-z) contains supplementary material, which is available to authorized users.

## Background

The latest European Perinatal Health Report identified 22.6% of German women as being overweight (body mass index (BMI) 25.0–29.9 kg/m^2^) and 13.7% as obese (BMI ≥ 30.0 kg/m^2^) at the onset of pregnancy [1]. Maternal overweight and obesity can affect the course of pregnancy, as well as delivery and the postpartum health of both mothers and their infants [2, 3]. In addition to a high pre-pregnancy BMI, excessive gestational weight gain (GWG) is an increasing public health concern due to its potential contribution to pregnancy and obstetric complications, maternal postpartum weight retention and childhood obesity. With reference to the recommendations for adequate weight gain during pregnancy provided by the United States’ Institute of Medicine (IOM) [4], there is a significant trend towards excessive GWG [5]. In Germany, more than 40% of pregnant women currently exceed the recommended weight gain thresholds [6].

Pregnant women who gain weight excessively are more likely to develop gestational diabetes mellitus (GDM) [7, 8] and to retain weight in the postpartum period [9]. These risks not only affect overweight women or those with obesity, but also those entering pregnancy with a normal BMI [8, 10, 11]. Further, high maternal weight gain in pregnancy has been found to increase the risk of high foetal birth weight and obstetric complications [12]. In addition, high GWG has been shown to raise the risk of childhood overweight and obesity [13, 14], especially in infants born to women with a normal pre-pregnancy BMI [15], which can persist later in life [16, 17].

Over the last decade, a variety of lifestyle intervention studies during pregnancy have tried to limit GWG and to improve maternal and infant health. However, these randomised controlled trials have shown rather modest effects in reducing excessive GWG and its associated health outcomes [18–21]. Nevertheless, several studies involving diet and/or physical activity counselling suggest effects on GWG, and a recent meta-analysis showed a mean reduction of GWG by 0.7 kg due to lifestyle interventions [22]. Yet, many lifestyle intervention trials have focused only on overweight and/or obese women, and trials recruiting women across the entire BMI range do not always explicitly evaluate the effectiveness in normal-weight women [23]. Further, only a limited number of small studies have tried to integrate lifestyle programmes into routine antenatal care outside of academic settings [24–26]. Thus, there remains a clear need to develop effective and efficient ‘real-world’ strategies limiting GWG to appropriate levels in women with normal weight, as well as in those overweight and with obesity.

We recently conducted the FeLIPO pilot trial (“Feasibility of a lifestyle intervention in pregnancy to optimize maternal weight development”) in order to evaluate the potential to prevent excessive GWG within the setting of routine prenatal care [27]. The intervention programme, which focused on a balanced diet, physical activity and self-monitoring of GWG, was delivered by an experienced dietician and was effective in reducing the proportion of women with excessive GWG according to the IOM recommendations (38% vs. 60%, *p* = 0.032) [27]. Based on the results of this pilot trial, the GeliS (“Gesund leben in der Schwangerschaft”/Healthy living in pregnancy) trial was designed to embody a true public health approach, as it was performed within the framework of the well-established German pre- and postnatal care system used by almost every pregnant woman in the country. The intervention offered comprehensive counselling on a healthy perinatal lifestyle at four defined visits and aimed to prevent excessive GWG and associated maternal and infant health outcomes.

## Methods

### Study design

The GeliS study is a public health project targeting maternal and infant health with individual lifestyle counselling during pregnancy. The study was designed as a prospective, multicentre, cluster-randomised, controlled, open intervention trial in five administrative regions of Bavaria, a federal state in south-eastern Germany. Within these five regions, paired cluster randomisation was conducted by matching two areas per region according to birth figures and socioeconomic status. In each of the five pairs, both urban and rural districts were included. One area of each pair was randomly assigned to the intervention and the other to the control group. As the study was conducted in gynaecological and midwifery practices, it depicts the real-life setting of routine prenatal care in Germany. A detailed description of the rationale, study design and methods has been previously published [28].

The study was conducted in accordance with the Declaration of Helsinki as well as with current local regulatory requirements and laws. The study protocol was approved by the ethics committee of the Technical University of Munich and registered at the ClinicalTrials.gov Protocol Registration System (NCT01958307).

### Participants

Women with a pre-pregnancy BMI of between ≥ 18.5 kg/m^2^ and ≤ 40.0 kg/m^2^ and a singleton pregnancy were recruited prior to the end of the 12^th^ week of gestation by medical personnel in gynaecological and midwifery practices. In order to represent the general healthy population, underweight women or those with severe obesity were excluded. Women were eligible if they were aged between 18 and 43 years, had sufficient German language skills and provided written informed consent. All women completed a short screening questionnaire to obtain demographic data and pre-pregnancy weight. Women with a multiple or complicated pregnancy and women with severe pre-existing illnesses were excluded, as previously described [28].

While participants in the control group (C) received general information leaflets on a healthy lifestyle in pregnancy and continued to attend routine prenatal care, participants of the intervention group (IV) additionally obtained a comprehensive lifestyle intervention programme.

### Lifestyle intervention programme

Pregnant women in the intervention group attended three individual face-to-face counselling sessions during the course of pregnancy (at 12–16, 16–20 and 30–34 weeks of gestation) and one after delivery at 6–8 weeks postpartum, each lasting 30–45 minutes. Sessions were delivered by previously trained midwives, gynaecologists or medical assistants from the participating gynaecological practices. The training consisted of two seminars with a total of approximately 10 hours of structured teaching. To ensure consistency in counselling practice and content, lifestyle counsellors obtained a presentation binder with easily understandable material to use in each counselling session as well as checklists for scheduling and documentation of sessions.

The counselling sessions were performed alongside routine pre- and postnatal visits in gynaecological and midwifery practices. Pregnant women were encouraged to consume a balanced diet, engage in physical activity and to self-monitor weight gain. For self-monitoring of GWG, women received a weight-gain chart according to their baseline BMI category, which depicted weight development during the course of pregnancy as recommended by the IOM [4, 28]. Information about a healthy diet in pregnancy was based on the recommendations of the Healthy Start – Young Family Network [29]. Next to the general principles of healthy nutrition, the relevance of critical nutrients (e.g. folate, iodine and iron) in pregnancy was addressed. The importance of avoiding alcohol and tobacco and of minimising the risk of food-borne infections were emphasized. Women were advised to achieve 150 minutes of moderate physical activity per week [30]. In order to facilitate adherence to physical activity recommendations, women received a brochure giving examples for appropriate exercise, a list of local prenatal exercise programmes and a pedometer to enable self-monitoring of daily physical activity. In addition to general information, individual feedback on dietary habits and physical activity, as assessed at study entry via questionnaires, was provided. Counsellors received checklists to compare behavioural parameters with recommendations in order to ensure consistency in gathering feedback. More details of the lifestyle programme are given in the published study protocol [28].

As a measure of process evaluation, several medical and midwifery practices were monitored to check if the intervention programme was performed as intended. For that purpose, a sample of lifestyle counselling sessions were supervised by a member of the study team who assessed the duration and delivery of content, as well as the use of study materials.

### Outcomes

The primary outcome of the study was measurement of the proportion of participating women who developed excessive GWG according to the IOM recommendations [4]. GWG was calculated as maternal weight at the last prenatal visit minus the weight measured at the first routine prenatal visit. Pre-pregnancy BMI calculation was based on self-reported pre-pregnancy weight at the time of recruitment. Weight was routinely measured in gynaecological or midwifery practices at every antenatal visit and was documented in the routinely used maternity records.

Secondary outcomes included the incidence of GDM, other pregnancy and obstetric complications, mode of delivery, anthropometric measures and health status of the newborns, as well as maternal weight retention (6–8 weeks postpartum). All data were extracted from maternity and birth records. For the screening and diagnosis of GDM, a standardised 2-hour 75 g oral glucose tolerance test (OGTT) was conducted at 24–28 weeks of gestation. In routine care, a non-fasting pre-test with 50 g of glucose is frequently applied, followed by a fasting 75 g test only in case of a measured blood glucose level ≥ 135 mg/dL (7.5 mmol/L) after 1 hour in the pre-test [31]. In the GeliS study, gynaecologists were instructed to directly perform the 75 g OGTT according to national and international guidelines [32, 33]. GDM was diagnosed if at least one of the following thresholds was equalled or exceeded: fasting plasma glucose: 92 mg/dL (5.1 mmol/L), 1-hour value: 180 mg/dL (10.0 mmol/L), and 2-hour value: 153 mg/dL (8.5 mmol/L). Depending on the severity, some of the women with a GDM diagnosis received treatment (dietary counselling or insulin treatment) as prescribed by the treating gynaecologists at their own discretion. However, every participating practice received information on the current national guidelines for the management of GDM. To obtain additional information on the presence and severity of GDM, glycated haemoglobin was measured at 30–34 weeks of gestation.

Prior to the data entry at the Munich Study Centre, all data were checked for quality and pseudonymised.

### Power calculation and statistical analysis

The study was designed to have 90% power to detect a between-group difference of 10% in the primary outcome, with a 5% significance level and an intraclass correlation coefficient of 0.5% [28, 34]. Power calculation was based on the proportion of women exceeding the IOM weight gain recommendations and the results of the pilot trial [27]. It was suggested that at least 40% of women (BMI ≥  18.5 kg/m^2^) in the control group would gain weight in excess [14]. A reduction of this proportion to 30% in the intervention group was expected. Power calculation suggested a sample size of 1900 women [35]. Considering a drop-out rate of up to 20% during pregnancy, as well as a potential imbalance in group size between the intervention and control groups, the recruitment of 2500 women was planned.

The primary outcome was compared between the intervention and control groups using logistic regression models fit using generalised estimating equations due to the cluster-randomised design [35]. Unadjusted and adjusted analyses were conducted, adjusting for pre-pregnancy BMI category, age, parity and gestational age at the first prenatal visit. Subgroup analyses according to pre-pregnancy BMI category were performed. The presented results correspond to complete-case analyses including all participants for whom primary outcome data were available, apart from those women who had a preterm delivery (< 37 weeks of gestation). Women with preterm delivery were excluded from the analysis in order to present the proportion of women with excessive GWG in a full-term pregnancy. In addition, multiple imputation using fully conditional specification was used to generate 10 imputed datasets with results pooled across these datasets [36]. Further, a pre-specified per-protocol analysis was performed excluding those participants whose weight was not measured at screening or last visit prior to birth, who violated inclusion/exclusion criteria or, for those in the intervention group, who missed a lifestyle counselling session or had a counselling session more than 2 weeks later than planned.

The secondary outcomes were similarly compared between intervention and control groups, with linear, logistic and multinomial generalised estimating equation models respectively fit for the continuous, binary and categorical outcome variables.

All analyses were performed using SAS software, version 9.4 for Windows (SAS Institute Inc., Cary, NC, USA).

## Results

### Flow-chart and baseline characteristics of participants

**Fig. 1:**
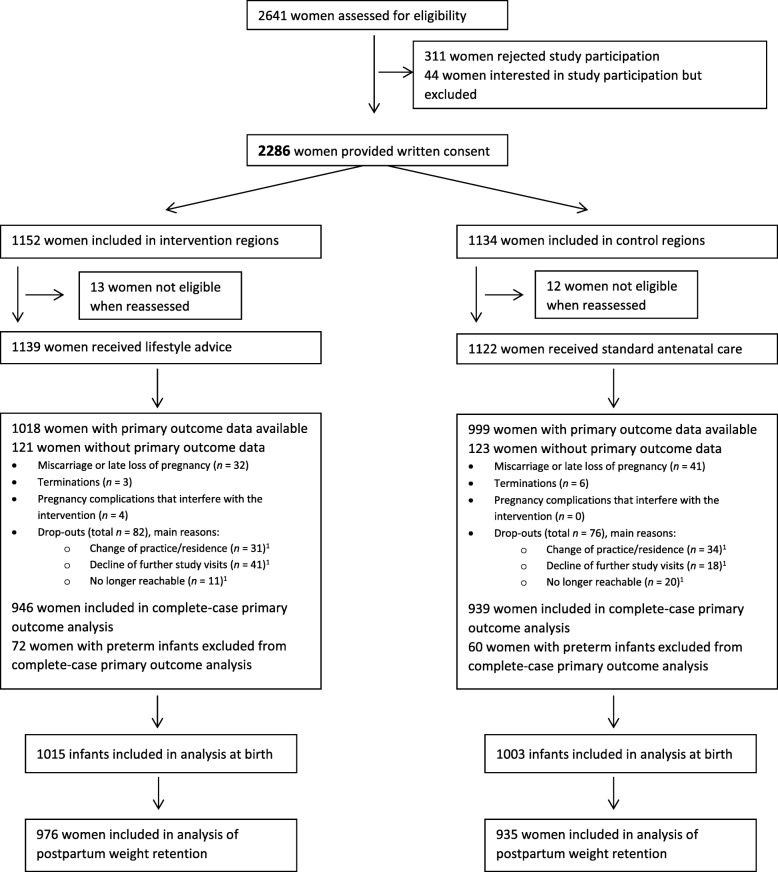
Flow-chart of the GeliS trial. ^1^multiple reasons possible; GeliS, “Gesund leben in der Schwangerschaft”/Healthy living in pregnancy

The flow of participants in the GeliS trial is presented in Fig. 1. Between the years 2013 and 2015, 2641 women were assessed for eligibility and 2286 were subsequently recruited for study participation. Of these, 25 women were not eligible when reassessed, 1139 women received lifestyle advice (delivered by 63 trained counsellors) and 1122 standard antenatal care in 71 participating practices (39 in the intervention regions and 32 in the control regions). Primary outcome data of 1885 women was included in the complete-case analysis. Reasons for missing outcome data were miscarriage (*n* = 73), termination (*n* = 9) or severe pregnancy complications (*n* = 4). A further 158 (7.0%) women dropped out from both groups due to (multiple answers were possible) change of practice or residence (*n* = 65), decline of further study visits (*n* = 59) or no longer reachable (*n* = 31). In total, 132 women were excluded from GWG analysis due to preterm delivery (Fig. 1). The data of 2018 children were included in the analyses. The low drop-out rate enabled recruitment to be stopped with a lower number of participants than the originally planned 2500 women.

An overview of baseline characteristics is given in Table 1. A total of 23.0% of included women were characterised as overweight and 12.1% with obesity. Mean self-reported weight and pre-pregnancy BMI were similar in the intervention and control groups (68.4 kg vs. 68.0 kg; 24.4 kg/m^2^ vs. 24.3 kg/m^2^). In the intervention group, more women were nulliparous (62.0% vs. 53.0%). Maternal age, gestational age at entry and educational level were comparable between the two groups.

**Table 1:** Baseline characteristics of study participants

	Intervention (*n* = 1139)	Control (*n* = 1122)	Total (*n* = 2261)
Age, years	30.2 ± 4.4^a^	30.4 ± 4.7	30.3 ± 4.5
Height, cm	167.4 ± 6.0	167.2 ± 6.0	167.3 ± 6.0
Pregravid weight, kg	68.4 ± 13.1	68.0 ± 13.7	68.2 ± 13.4
Pregravid BMI, kg/m^2^	24.4 ± 4.4	24.3 ± 4.6	24.4 ± 4.5
Pregravid BMI category, n (%)			
BMI 18.5–24.9 kg/m^2^	732/1139 (64.3%)	735/1122 (65.5%)	1467/2261 (64.9%)
BMI 25.0–29.9 kg/m^2^	271/1139 (23.8%)	249/1122 (22.2%)	520/2261 (23.0%)
BMI 30.0–40.0 kg/m^2^	136/1139 (11.9%)	138/1122 (12.3%)	274/2261 (12.1%)
Gestational age at entry, weeks	8.1 ± 2.1	8.4 ± 2.2	8.3 ± 2.2
Nulliparous, n (%)	706/1139 (62.0%)	593/1119 (53.0%)	1299/2258 (57.5%)
Current smoker, n (%)	60/1061 (5.7%)	64/1044 (6.1%)	124/2105 (5.9%)
Number of cigarettes per day (smokers)	4.6 ± 3.1	6.2 ± 4.4	5.4 ± 3.9
Previous caesarean section	105/1027 (10.2%)	117/1013 (11.5%)	222/2040 (10.9%)
Previous preterm birth	26/1027 (2.5%)	30/1013 (3.0%)	56/2040 (2.7%)
Country of birth, n (%)			
Germany	1001/1138 (88.0%)	1001/1118 (89.5%)	2002/2256 (88.7%)
Other	137/1138 (12.0%)	117/1118 (10.5%)	254/2256 (11.3%)
Living with a partner	1093/1134 (96.4%)	1065/1118 (95.3%)	2158/2252 (95.8%)
Educational level			
None	2/1138 (0.2%)	4/1115 (0.4%)	6/2253 (0.3%)
General secondary school	172/1138 (15.1%)	182/1115 (16.3%)	354/2253 (15.7%)
Intermediate secondary school	486/1138 (42.7%)	466/1115 (41.8%)	952/2253 (42.3%)
High school/Grammar school	478/1138 (42.0%)	463/1115 (41.5%)	941/2253 (41.8%)
University degree	297/1132 (26.2%)	296/1105 (26.8%)	593/2237 (26.5%)

^a^Mean ± SD (all such values)

*BMI* body mass index

### Gestational weight gain

**Fig. 2:**
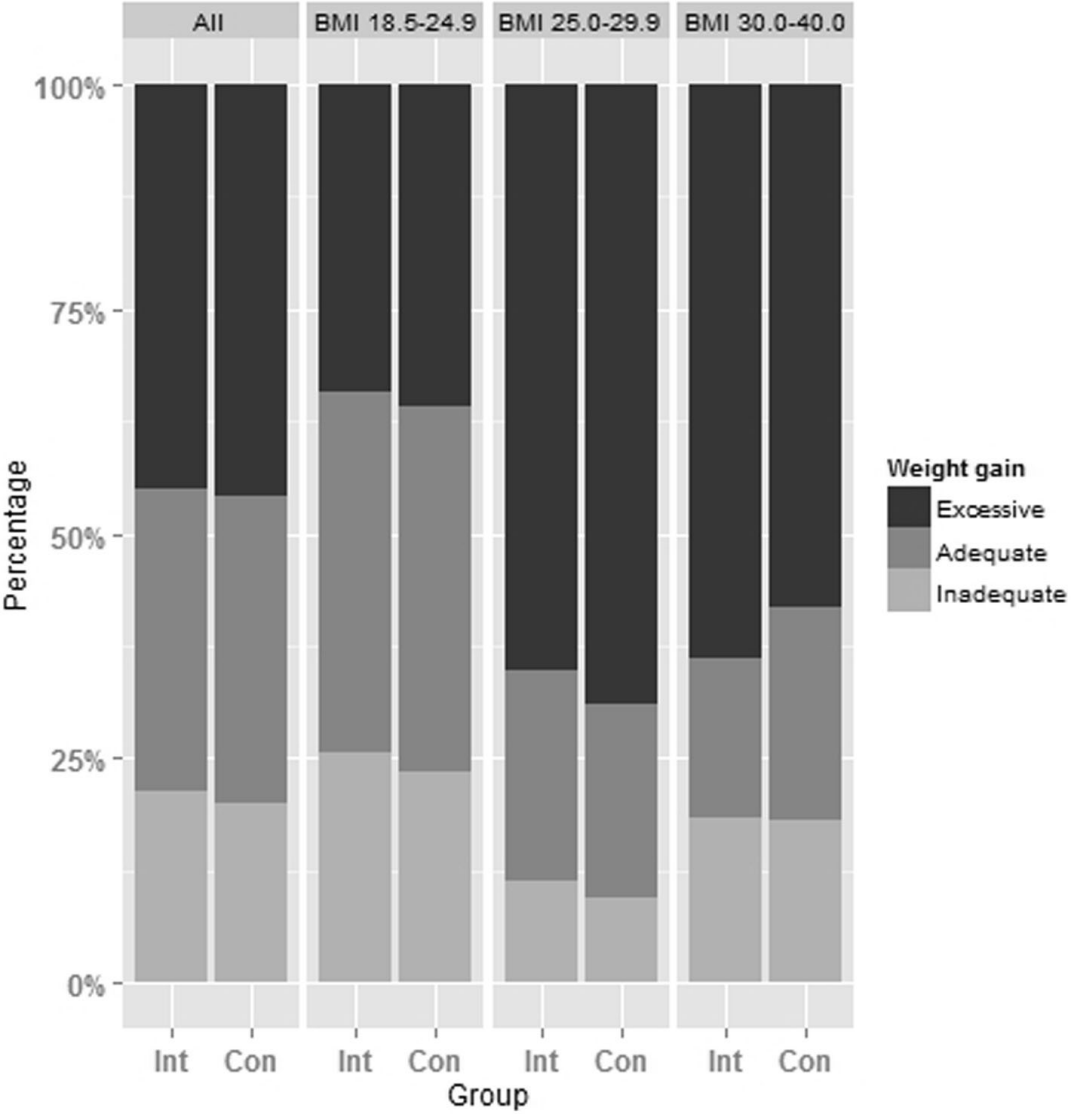
Inadequate, adequate and excessive gestational weight gain (GWG) among women with different body mass index (BMI, kg/m^2^) categories. Inadequate, adequate and excessive GWG is defined according to the criteria provided by the U.S. Institute of Medicine [4]. *Con* control group, *Int* intervention group

Total weight gain and the proportion of women exceeding the IOM weight gain recommendations are shown in Table 2. The IOM recommendations were exceeded by 45.1% and 45.7% of women in the intervention and control groups, respectively. There was no statistically significant evidence of a difference between groups (adjusted odds ratio 0.95, 95% confidence interval (CI) 0.66 to 1.38, *p* = 0.789). The intraclass correlation coefficient, reflecting potential systematic differences between the clustered study regions, was low (0.8%). Similar results for the primary outcome were obtained in the per-protocol and the multiple imputation analyses (Additional file 1: Table S1). Subgroup analyses according to pre-pregnancy BMI also provided no evidence of differences in excessive GWG between the intervention and control groups. Mean total GWG was 14.7 kg in women with normal pre-pregnancy weight, 14.0 kg in overweight women and 11.0 kg in women with obesity, and did not significantly differ between routine care and intervention group (adjusted estimated mean difference for BMI groups combined 0.1 kg, 95% CI – 0.8 to 1.0 kg, *p* = 0.838). Adherence to IOM recommendations according to pre-pregnancy BMI subgroups (Fig. 2) showed that excessive GWG was more frequent among overweight women (IV: 65.2% and C: 69.0%) and those with obesity (IV: 63.9% and C: 58.3%) compared to normal weight participants (IV: 34.2% and C: 35.9%). In total, 21.4% in the intervention and 19.9% in the control group gained weight below IOM recommendations.

**Table 2:** Excessive and mean gestational weight gain in lifestyle intervention and control groups

	Intervention (*n* = 946)	Control (*n* = 939)	Absolute effect size (95% CI)	*p* value	Adjusted effect size^a^ (95% CI)	Adjusted *p* value^a^
Women with excessive GWG (> IOM)	427/946 (45.1%)	429/939 (45.7%)	1.05 (0.76 to 1.43)	0.779	0.95 (0.66 to 1.38)	0.789
BMI 18.5–24.9 kg/m^2^	208/608 (34.2%)	224/624 (35.9%)	1.00 (0.68 to 1.47)	0.988	0.92 (0.61 to 1.38)	0.674
BMI 25.0–29.9 kg/m^2^	150/230 (65.2%)	138/200 (69.0%)	0.85 (0.55 to 1.33)	0.478	0.81 (0.51 to 1.29)	0.382
BMI 30.0–40.0 kg/m^2^	69/108 (63.9%)	67/115 (58.3%)	1.27 (0.75 to 2.14)	0.375	1.08 (0.62 to 1.87)	0.790
Total weight gain, kg	14.1 ± 5.3^b^	14.1 ± 5.2	0.28 (– 0.61 to 1.17)	0.542	0.09 (– 0.79 to 0.97)	0.838
BMI 18.5–24.9 kg/m^2^	14.6 ± 4.5	14.8 ± 4.6	0.06 (– 0.79 to 0.90)	0.899	– 0.10 (– 0.93 to 0.72)	0.808
BMI 25.0–29.9 kg/m^2^	14.0 ± 6.0	14.1 ± 5.5	– 0.02 (– 0.90 to 0.86)	0.958	– 0.26 (– 1.14 to 0.63)	0.569
BMI 30.0–40.0 kg/m^2^	11.5 ± 6.8	10.6 ± 6.5	1.00 (– 0.90 to 2.90)	0.301	0.52 (– 1.05 to 2.09)	0.513

^a^Adjusted for pre-pregnancy BMI, age, parity and gestational age at first visit

^b^Mean ± SD (all such values)

*BMI* body mass index, *GWG* gestational weight gain, *IOM* Institute of Medicine [4]

### Maternal and obstetric outcomes

There were no major differences in maternal outcomes between groups (Table 3). Gestational diabetes mellitus was diagnosed in 10.8% of women allocated to the lifestyle intervention and 11.1% of women in the control group (adjusted odds ratio 0.84, 95% CI 0.41 to 1.71, *p* = 0.622). GDM treatment was administered to 3.5% and 4.9% of women in the intervention and the control groups, respectively. Mean glycated haemoglobin in late pregnancy was 32.2 mmol/mol (5.1%) and 32.1 mmol/mol (5.1%) in the intervention and control groups, respectively. Elevated blood pressure was reported more often in the intervention group (IV: 9.5% and C: 6.4%, *p* = 0.017). Mean short-term weight retention at 6–8 weeks postpartum was 4.0 ± 4.8 kg in women receiving lifestyle counselling and 4.3 ± 4.8 kg in the standard care group (*p* = 0.649). The intervention did not increase the risk of complications such as vaginal bleeding or preterm labour (Table 3).

**Table 3:** Maternal outcomes in lifestyle intervention and control groups

	Intervention	Control	Unadjusted effect size (95% CI)	*p* value	Adjusted effect size^a^ (95% CI)	Adjusted *p* value^a^
Pregnancy complications						
Gestational diabetes mellitus	109/1008 (10.8%)	106/954 (11.1%)	0.74 (0.37 to 1.47)	0.383	0.84 (0.41 to 1.71)	0.622
Dietary treatment	24/1008 (2.4%)	33/954 (3.5%)				
Insulin treatment	19/1008 (1.8%)	17/954 (1.8%)				
Treated GDM	35/1008 (3.5%)	47/954 (4.9%)	0.68 (0.33 to 1.39)	0.285	0.76 (0.43 to 1.37)	0.364
HbA_1c_, mmol/mol	32.2 ± 3.7	32.1 ± 3.5	0.15 (– 0.45 to 0.74)	0.633	0.24 (– 0.40 to 0.87)	0.468
HbA_1c_, %	5.1 ± 0.3	5.1 ± 0.3	0.01 (– 0.00 to 0.03)	0.126	0.02 (– 0.01 to 0.05)	0.119
Hypertension^b^	99/1041 (9.5%)	66/1039 (6.4%)	1.56 (0.98 to 2.49)	0.060	1.64 (1.09 to 2.45)	0.017
Preeclampsia/HELLP syndrome	14/1006 (1.4%)	13/965 (1.3%)	c		c	
Bleeding	31/956 (3.2%)	39/893 (4.4%)	0.69 (0.35 to 1.34)	0.270	0.65 (0.32 to 1.29)	0.216
Preterm labour	15/956 (1.6%)	26/893 (2.9%)	0.54 (0.35 to 0.84)	0.006	0.51 (0.33 to 0.79)	0.003
Obstetric outcomes						
Birth mode						
Spontaneous birth	615/1016 (60.5%)	628/1003 (62.6%)	Reference		Reference	
Elective caesarean section	157/1016 (15.5%)	117/1003 (11.6%)	1.37 (1.05 to 1.78)	0.019	1.41 (1.08 to 1.85)	0.017
Emergency caesarean section	150/1016 (14.8%)	159/1003 (15.9%)	0.96 (0.75 to 1.24)	0.769	0.86 (0.67 to 1.12)	0.257
Instrumental vaginal delivery	94/1016 (9.3%)	99/1003 (9.9%)	0.97 (0.72 to 1.31)	0.842	0.83 (0.61 to 1.14)	0.245
Induction of labour	174/1007 (17.3%)	233/992 (23.5%)	0.71 (0.48 to 1.06)	0.098	0.65 (0.43 to 0.98)	0.038
Anaesthesia	769/1001 (76.8%)	787/983 (80.1%)	1.05 (0.55 to 2.00)	0.871	1.23 (0.69 to 2.19)	0.472
Weight retention, kg (6–8 weeks postpartum)	4.0 ± 4.8^d^ (*n* = 976)	4.3 ± 4.8 (*n* = 934)	– 0.13 (– 0.95 to 0.69)	0.760	– 0.19 (– 1.01 to 0.63)	0.649

^a^Adjusted for pre-pregnancy BMI, age and parity

^b^Systolic blood pressure > 140 mmHg or diastolic blood pressure > 90 mmHg at at least two time points

^c^No statistic testing due to small number of cases

^d^Mean ± SD (all such values)

*GDM* gestational diabetes mellitus, *HbA1c* glycated haemoglobin

Most obstetric outcomes did not significantly differ between the two groups. However, elective and emergency caesarean section rates were 15.5% and 14.8%, respectively, in the intervention group and 11.6% and 15.9%, respectively, in the control group. Further, labour was induced more often (IV: 17.3% vs. C: 23.5%, *p* = 0.038) and preterm labour was more frequently reported (IV: 1.6% vs. C: 2.9%, *p* = 0.003) in the control group.

### Neonatal outcomes

Neonatal outcomes are summarised in Table 4. Mean birth weight and length were slightly lower in the intervention group (weight: 3313 ± 536 g vs. 3363 ± 498 g, adjusted estimated mean difference – 44.0 g, 95% CI – 81.0 to – 7.0 g, *p* = 0.020; length: 51.1 ± 2.7 cm vs. 51.6 ± 2.5 cm, adjusted estimated mean difference – 0.5 cm, 95% CI – 0.7 to – 0.2 cm, *p* = 0.001). The proportion of neonates born large for gestational age (LGA) and small for gestational age did not significantly differ between groups. The rate of preterm births was low in both groups (IV: 7.1% vs. C: 6.0%). One stillbirth was observed in the intervention group and one neonatal death was recorded in each group. The intervention did not lead to a significant increase in neonatal complications at birth such as adjustment disorders, cardiac irregularities or hypoglycaemia (10.0% vs. 8.2%, *p* = 0.310; Table 4).

**Table 4:** Neonatal outcomes in lifestyle intervention and control groups

	Intervention	Control	Unadjusted effect size (95% CI)	*p* value	Adjusted effect size^a^ (95% CI)	Adjusted *p* value^a^
Birth weight, g	3313 ± 536^b^ (*n* = 1015)	3363 ± 498 (*n* = 1003)	– 50.2 (– 80.6 to – 19.7)	0.001	– 44.0 (– 81.0 to – 7.0)	0.020
Birth length, cm	51.1 ± 2.7 (*n* = 1011)	51.6 ± 2.5 (*n* = 992)	– 0.5 (– 0.7 to – 0.2)	< 0.001	– 0.5 (– 0.7 to – 0.2)	0.001
Head circumference, cm	34.6 ± 1.7 (*n* = 1008)	34.8 ± 1.5 (*n* = 973)	– 0.2 (– 0.4 to 0.0)	0.070	– 0.2 (– 0.4 to 0.0)	0.093
Large for gestational age (> 90th percentile)	73/1013 (7.2%)	75/1003 (7.5%)	0.99 (0.87 to 1.12)	0.869	1.01 (0.86 to 1.20)	0.864
Small for gestational age (< 10th percentile)	88/1013 (8.7%)	84/1003 (8.4%)	1.06 (0.83 to 1.34)	0.651	1.03 (0.82 to 1.31)	0.780
Macrosomia (weight > 4500 g)	13/1015 (1.3%)	6/1003 (0.6%)	2.01 (0.58 to 6.89)	0.268	2.28 (0.66 to 7.90)	0.195
Preterm birth	72/1014 (7.1%)	60/1004 (6.0%)	1.21 (0.83 to 1.77)	0.319	1.18 (0.78 to 1.79)	0.437
Neonatal complications at birth	101/1014 (10.0%)	82/1001 (8.2%)	1.23 (0.90 to 1.68)	0.186	1.18 (0.86 to 1.61)	0.310

^a^Adjusted for pre-pregnancy BMI, age and parity

^b^Mean ± SD (all such values)

### Process evaluation

The first planned appointment (at 12–16 weeks of gestation) was attended by 98.3% of women in the intervention group, the second (16–20 weeks of gestation) by 97.6%, the third (30–34 weeks of gestation) by 95.9%, and the postpartum session by 93.7% (Additional file 2: Table S2). In total, 87.6% of women visited all four appointments and 2.7% did not attend any counselling session. The mean number of attended sessions was 3.7. During pregnancy, 2.8 sessions were attended on average. The majority of sessions were performed in the correct time intervals (Additional file 2: Table S2).

The supervised counselling sessions had a median duration of 35 minutes (*n* = 53) (Additional file 3: Table S3). The presentation binder containing specific counselling content was used in 86.8% of monitored sessions. In 69.6% of these cases, all the predefined counselling content was discussed. Weight monitoring with the provided weight-gain chart and individual counselling were performed in 73.6% and 62.3% of supervised sessions, respectively (Additional file 3: Table S3).

## Discussion

The results of the GeliS study suggest that providing lifestyle advice addressing diet, physical activity and weight monitoring within routine care during pregnancy is not effective in avoiding excessive GWG or in reducing total GWG. Except for a slight decrease in birth weight and length, the antenatal intervention did not notably affect the risk of developing GDM or any other maternal or foetal outcome.

The GeliS trial was performed as a public health approach in the real-life setting of routine care. As researchers have previously suggested [37, 38], study procedures were adapted to the daily routine work of gynaecological and midwifery practices in order to allow an easy implementation of the intervention into the German maternity healthcare system. To this end, lifestyle counselling was conducted by previously trained medical personnel.

To our knowledge, there is no other trial to date that has investigated the compatibility of an additional lifestyle advisory component within routine healthcare for pregnant women to such a large extent. Overall, 45.1% of women who received the intervention exceeded the IOM recommendations, compared to 45.7% of women who received standard antenatal care. The lack of an intervention effect is consistent with current research. Despite an intensive intervention programme of eight group sessions, the UPBEAT trial showed only small effects on GWG in pregnant women with obesity (– 0.55 kg) [21]. A large meta-analysis of lifestyle intervention trials including individual participant data of more than 12,000 pregnant women reached similar conclusions [22]. According to the i-WIP collaborative group [22], lifestyle interventions are able to reduce GWG, but the effect was quite small (– 0.7 kg), and 37% of women still exceeded the weight gain recommendations. Standard care differs between countries and, thus, may influence the effectiveness of different interventions [39]. Moreover, the effect of lifestyle interventions in different groups of women based on BMI category, age, ethnicity, parity and risk status in pregnancy is not clear [37], which complicates the comparability between studies. Furthermore, the assessment of excessive GWG according to the IOM recommendations is disputed as evidence to support these guidelines and has been suggested as insufficient [40]. Especially in overweight women and those with obesity, weight gain within the IOM recommendations has been associated with both positive and adverse pregnancy outcomes [40]. Nevertheless, the IOM guidelines represent the current standard and are frequently applied [41].

As more than 40% of pregnant women in Germany and elsewhere exceed these recommendations, resulting in potentially adverse short- and long-term consequences for mothers and infants, there is an urgent demand for successful interventions [4, 6, 14, 42]. Additionally, pregnant women themselves are demanding lifestyle counselling, as determined by the high compliance to the GeliS intervention programme in terms of attendance to the scheduled sessions. More than 85% of women in the intervention group attended all four counselling appointments, showing their willingness to adhere to a healthy lifestyle. Together with high rates of excessive GWG, this emphasizes the gap between the current standard of prenatal care and the need for information and support.

Among the secondary outcome parameters, GDM was diagnosed with a 2-hour OGTT in the GeliS trial. At least one of the GDM diagnostic threshold values [33] was exceeded by 11.0% of the tested study participants, although this was slightly lower than the overall prevalence estimate for Germany (13.2%) [31]. As an important finding, the GeliS intervention did not lead to a reduction in GDM. However, most women had an adequate metabolic control, as assessed by the measurement of glycated haemoglobin, and only a minority required active treatment. This result is in line with observations from recent meta-analyses and reviews [22, 43, 44].

Despite most maternal and neonatal outcomes being unaffected by the intervention, a few differences were observed. Elective caesarean sections were more frequently reported in the intervention group. As mode of delivery was not addressed during lifestyle counselling, a specific effect of the intervention seems to be unlikely. Due to the cluster randomisation, one possible explanation could be differences in procedures between hospitals in performing caesarean sections, which is underpinned by a high variance in the caesarean section rate between regions [45]. Further, labour had to be induced more often in the control group than in the intervention group. Labour is induced frequently, especially after the estimated due date, but as gestational week at birth was comparable between groups, the difference in the rate of labour induction could again be related to clinic-specific procedures. Differences in hypertension cannot be attributed to the intervention programme since blood pressure measurement procedures may differ between the single practices. Another difference between groups was the proportion of women with preterm labour, which was slightly higher in the control group. This parameter was evaluated as a safety control in order to ensure that encouraging women to engage in physical activity during pregnancy would not lead to premature contractions. Thus, the finding that the proportion of preterm labour was even lower in the intervention group supports the safety of the physical activity component.

The intervention resulted in significant trends towards a lower birth weight and lower birth length in the intervention group. However, the estimated differences between groups were small. Overall, there is insufficient evidence for an effect of lifestyle interventions in pregnancy on neonatal birth weight [23]. The observed difference in birth length in the intervention group may explain the difference in birth weight. Nevertheless, there were no significant differences regarding infants born LGA, in line with the results of the LIMIT and UPBEAT trials, which also showed no effect of lifestyle advice on the number of infants born LGA [19, 21]. Similarly, the i-WIP consortium did not report any significant effects for neonates including birth weight [22].

Despite the lack of observation of major effects from the GeliS intervention on GWG or pregnancy complications, the trial has several strengths. A major advantage is that counselling sessions could be scheduled in combination with prenatal visits, resulting in both high participation and low drop-out rates. The drop-out rate of 11% was lower than the expected rate of up to 20%, reflecting the applicability of the programme and, indirectly, the interest of women to participate in the lifestyle programme; similar observations have been reported [46]. Moreover, the cluster-randomised design counteracted the spill-over effects of lifestyle counselling content from women in the intervention group to those in the control group, which is a further strength of the GeliS trial. Additionally, compared to available national data, the characteristics of women participating in the GeliS study were representative of the target population with respect to age, pre-pregnancy BMI and smoking status.

However, there are a few limitations worth noting. Women participating in the GeliS trial were predominantly white and relatively well educated, with only a small proportion from ethnic minorities. Therefore, the results may not be completely applicable to the general population of Germany. Moreover, the counselling was not extensively based on concepts of behaviour change, with lifestyle counselling including methods such as self-monitoring and feedback on behaviour. An extension with additional methods such as motivational interviewing was not possible. As discussed by others, intervention programmes are often initiated too late [20, 38]. The importance of implementing an antenatal intervention early on should be stressed since many women gain much weight in early pregnancy and high early GWG is strongly predictive not only of total excessive GWG [47], but also of GDM [8]. In accordance with these findings, GeliS counsellors were encouraged to include expectant mothers as early as possible.

It is noteworthy that the large majority of previous lifestyle intervention studies in pregnant women has been performed by trained experts within academic study centres, which poorly reflect real-life settings. Controlled studies involving exercise groups, objective monitoring of physical activity and continuous observation of dietary behaviour may be more likely to prevent excessive GWG. However, there is an urgent need to develop and evaluate effective strategies in real-life settings of routine prenatal care in order to be applicable at the population level. Apart from our pilot trial, of the two identified studies integrating lifestyle interventions into prenatal care [24, 25], only one was effective in reducing GWG [25]. Although this study was integrated into routine care, counselling was performed by a qualified expert instead of trained medical personnel as in the present study. This is in line with the results of our pilot trial, supporting the effectiveness of lifestyle counselling within routine care when performed by an expert [27]. Unfortunately, the promising results observed in our pilot study FeLIPO (excessive GWG IV: 38.2% vs. C: 59.5%) could not be confirmed through the GeliS intervention, despite the comparability of the GeliS trial in terms of trial setting and counselling content. However, in the FeLIPO trial, counselling was provided by a dietician, while trained medical personnel delivered the sessions in the GeliS study, which may have substantially contributed to the discrepancy of results.

Although counselling sessions in the GeliS trial were conducted according to a predefined curriculum, differences in the quality of the delivered intervention are the most likely explanation for the lack of effect on GWG. Due to the specific characteristics of the study designed as a public health approach, it was not possible to extensively monitor whether the sessions were consistently performed by the counsellors as planned, with only a sample of sessions being supervised by a member of the study team. Indeed, the process evaluation of this sample showed inconsistencies in the delivery of counselling sessions. Not every lifestyle counsellor addressed all planned components of the intervention. In particular, individual feedback based on personal dietary and physical activity habits was not consistently given. To date, the prenatal care provided by gynaecologists and midwives does not include lifestyle advice, and mainly focuses on foetal growth parameters and maternal and foetal complications. Even though counsellors received specific training prior to the intervention sessions and reported feeling adequately trained, a 2-day seminar may not be enough to qualify gynaecologists, medical assistants and midwives as specialists with sufficient expertise for high-quality lifestyle coaching.

A further contributor to the missing effect could be the general issue of scale-up of intervention studies following a promising pilot phase [24, 48, 49]. Implementing successful interventions in health care systems remains a challenge [48, 49]. Finally, the lack of statistical differences in study outcomes between the intervention and control groups may be attributed to increased awareness of a healthy lifestyle and behaviour change during pregnancy among women in the control group following reading of the study material and questionnaires on dietary behaviour and physical activity.

Implementing a lifestyle programme into daily work and combining counselling sessions with routine care visits remains a challenge. Therefore, collaboration between medical practices and specially trained and experienced dieticians or lifestyle coaches could lead to an improvement in the quality of lifestyle counselling, and may reduce the proportion of women with excessive GWG and associated health consequences. Additionally, ensuring an appropriate environment, such as a separate room designated for counselling patients, rather than conducting sessions in a busy practice office, could contribute to increased quality of the lifestyle intervention. Finally, a more extensive intervention programme comprising more than four counselling sessions may be more successful in promoting significant lifestyle changes.

## Conclusions

In the setting of routine prenatal care, lifestyle advice given by trained healthcare providers was not successful in limiting GWG. Nevertheless, the potential long-term effects of the intervention remain to be seen. Analysis of dietary behaviour and physical activity will provide further insights into the adherence of study participants to lifestyle advice and their relation to health outcomes, and may highlight certain components of the counselling content that may be emphasised in future trials. A follow-up observation until the fifth year of life will extensively evaluate further weight development as well as the long-term health of mothers and their infants.

## Additional file

Additional file 1: **Table S1.** Per-protocol and multiple imputation analyses of excessive gestational weight gain. (PDF 84 kb)

Additional file 2: **Table S2.** Quantitative evaluation of lifestyle counselling sessions (PDF 162 kb)

Additional file 3: **Table S3.** Qualitative evaluation of lifestyle counselling sessions (PDF 82 kb)

## Abbreviations

BMI: body mass index
C: control group
CI: confidence interval
GDM: gestational diabetes mellitus
GeliS: “Gesund leben in der Schwangerschaft”/Healthy living in pregnancy
GWG: gestational weight gain
IOM: Institute of Medicine
IV: intervention group
LGA: large for gestational age
OGTT: oral glucose tolerance test
