# Supplementary material for: Effects of a lifestyle intervention during pregnancy to prevent excessive gestational weight gain in routine care – the cluster-randomised GeliS trial
Source: BMC Med. 2019 Jan 14;17:5. doi: 10.1186/s12916-018-1235-z (PMC6330753; doi:10.1186/s12916-018-1235-z)
Supplement: Supplementary file 1 — Table S1. Per-protocol and multiple imputation analyses of excessive gestational weight gain. (PDF 84 kb) [file 12916_2018_1235_MOESM1_ESM.pdf]

Supplemental Table 1: Per-protocol and multiple imputation analyses of excessive gestational weight gain

	Control	Intervention	Absolute effect size[1] (95% CI)	p value[1]	Adjusted effect size[2] (95% CI)	Adjusted p value[2]
**Women with excessive GWG (>IOM)**						
Per protocol	421/905 (46.5%)	401/883 (45.4%)	1.02(0.75,1.37)	0.918	0.92(0.65,1.29)	0.628
Multiple imputation			1.03(0.77,1.39)	0.824	0.95(0.68,1.33)	0.754

[1]logistic regression models fit using generalized estimating equations
[2]logistic regression models fit using generalized estimating equations adjusted for pre-pregnancy BMI, age, parity and gestational age at 1st visit
GWG, gestational weight gain; IOM, Institute of Medicine
