# Supplementary material for: Effects of a lifestyle intervention during pregnancy to prevent excessive gestational weight gain in routine care – the cluster-randomised GeliS trial
Source: BMC Med. 2019 Jan 14;17:5. doi: 10.1186/s12916-018-1235-z (PMC6330753; doi:10.1186/s12916-018-1235-z)
Supplement: Supplementary file 2 — Table S2. Quantitative evaluation of lifestyle counselling sessions (PDF 162 kb) [file 12916_2018_1235_MOESM2_ESM.pdf]

Supplemental Table 2: Quantitative evaluation of lifestyle counselling sessions

	**Number of participating women (intervention group)**	**Correct time interval**
**Individual counselling sessions**		
Session 1: 12th-16th week of gestation	1069/1087[1] (**98.3%**)	953/1069 (**89.1%**)
Session 2: 16th-20th week of gestation	1047/1073[1] (**97.6%**)	957/1047 (**91.4%**)
Session 3: 30th-34th week of gestation	1006/1049[1] (**95.9%**)	855/1006 (**85.0%**)
Session 4: 6-8 weeks postpartum	974/1039[1] (**93.7%**)	759/974 (**77.9%**)
**Number of counselling sessions**		
No session	30/1099[2] (**2.7%**)	
One session	21/1099[2] (**1.9%**)	
Two sessions	32/1099[2] (**2.9%**)	
Three sessions	54/1099[2] (**4.9%**)	
Four sessions	963/1099[2] (**87.6%**)	

[1]number of women in the intervention group at the time of the respective session
[2]number of women in the intervention group excluding women with major pregnancy complications
