# Supplementary material for: Effects of a lifestyle intervention during pregnancy to prevent excessive gestational weight gain in routine care – the cluster-randomised GeliS trial
Source: BMC Med. 2019 Jan 14;17:5. doi: 10.1186/s12916-018-1235-z (PMC6330753; doi:10.1186/s12916-018-1235-z)
Supplement: Supplementary file 3 — Table S3. Qualitative evaluation of lifestyle counselling sessions (PDF 82 kb) [file 12916_2018_1235_MOESM3_ESM.pdf]

Supplemental Table 3: Qualitative evaluation of lifestyle counselling sessions

**Quality parameters**	
Monitored counselling sessions (n)	53
Median duration of sessions (min)	35
Utilisation of study documents (n)	46/53 (**86.8%**)
Among study document users: completeness of counselling contents (n)	32/46 (**69.6%**)
Weight monitoring with weight gain chart (n)	39/53 (**73.6%**)
Individual counselling (n)	33/53 (**62.3%**)
